# PRMT5 as a Key Driver of Stemness and Metastatic Potential in Triple-Negative Breast Cancer

**DOI:** 10.3390/biom16060916

**Published:** 2026-06-20

**Authors:** Jae Jin Jeong, Mauli Maniar, Shahrzad Ghane, Sakshi Deshpande, Claire Ellis, Ashakumary Lakshmikuttyamma

**Affiliations:** Department of Pharmaceutical Sciences, Jefferson College of Pharmacy, Thomas Jefferson University, Philadelphia, PA 19107, USA

**Keywords:** epigenetics, PRMT5, breast cancer, TNBC

## Abstract

Protein arginine methyltransferase 5 (PRMT5) mediates arginine methylation of a wide range of proteins and plays context-dependent oncogenic or tumor-suppressive roles. In cancer, PRMT5 represses several tumor suppressor genes, including E-cadherin, TP53BP1, ST7, PTEN, and RB (retinoblastoma). Elevated PRMT5 expression has been reported across multiple cancer types, notably triple-negative breast cancer (TNBC). In TNBC, high PRMT5 levels are associated with enhanced cancer stem cell self-renewal, increased tumor growth and metastasis, and reduced patient survival. Mechanistically, PRMT5 promotes breast cancer stem cell maintenance and proliferation through stabilization of the transcription factors KLF4 and KLF5. Disruption of the PRMT5–KLF4 axis results in significant tumor reduction in TNBC models. Moreover, increased PRMT5 expression has been linked to resistance to chemotherapy and immunotherapy in TNBC. Notably, PRMT5 inhibitors demonstrate synergistic anticancer activity when combined with inhibitors of key oncogenic signaling pathways, including EGFR, PARP, and AKT. While several PRMT5 inhibitors are currently being evaluated in clinical trials for other malignancies, no clinical trials have yet been initiated specifically for TNBC.

## 1. Introduction

The major epigenetic changes are chromatin remodeling, DNA methylation, and histone protein modifications. Modifications at the N-terminal histone tails are considered post-translational protein modifications. Methylation, acetylation, and phosphorylation are the major histone modifications [1,2,3]. Histone methylation commonly happens on the lysine and arginine side chains. Histone methylation at the lysine residue may be mono, di, or tri-methylated, whereas mono or di-methylation occurs at the arginine residue [4,5]. Histone acetylation occurs at lysine residues, and the two major enzymes involved in the process are histone acetyltransferases (HATs) and histone deacetylases (HDACs) [4].

## 2. Protein Arginine Methyltransferase (PRMT)

The protein arginine methyltransferase (PRMT) family of enzymes catalyzes protein arginine methylation. Three classes of arginine methyltransferase exist. Type I generates monomethyl arginine (Rme1) and asymmetric dimethylarginine (Rme2a) [4]. Type II is involved in the production of Rme1 and symmetric dimethylarginine (Rme2s). Type III produces Rme1 only. Type I PRMTs are PRMT-1, 2, 3, 4, 6, and 8; type II PRMTs are PRMT-5 and 9; and PRMT7 is the only type III enzyme [4]. PRMTs catalyze the transfer of a methyl group from S-adenosyl methionine (SAM) to the ω- guanidine nitrogen (ω-NG) of a peptidyl arginine residue [4,6,7].

PRMTs catalyze the transfer of methyl groups to arginine residues on both histone and nonhistone proteins from S-adenosylmethionine (SAM). This epigenetic change leads to chromatin modification and increases DNA accessibility. It influences other post-translational modifications including lysine methylation, acetylation, and phosphorylation. It also regulates DNA methylation, RNA polymerase II (Pol II) and various transcription factor methylation [8,9]. Further, variations in gene transcriptional regulations by different PRMTs have been reported. Among PRMTs, 85% of mammalian cells express PRMT1, which is involved in various signaling pathways, including cell proliferation and apoptosis [9]. PRMT2 increases the transcriptional action of multiple hormone receptors. PRMT3 is involved in the methylation of 40S ribosomal protein S2 (rpS2) and 80S ribosome proper maturation. PRMT4 (CARM1-coactivator-associated arginine methyltransferase) increases the transcriptional activation by several nuclear hormone receptors [6,7].

### 2.1. PRMT5

PRMT5 is involved in the methylation of various proteins, including histones H2A, H3, and H4, as well as many cellular receptors [10]. The downstream signaling molecules regulated by PRMT5 may exert either oncogenic or tumor-suppressive effects. PRMT5 dimethylates H4R3me2s and H3R8me2s, creating more compact chromatin that suppresses gene expression. Studies showed that PRMT5-mediated H4R3me2s regulate DNA methylation by recruiting DNMT3A [11]. CDH1 (E-cadherin), TP53BP1, ST7, PTEN, and RB (retinoblastoma) are repressed genes in cancers by PRMT5-mediated repression. PRMT5 is also involved in the transcriptional activation of different oncogenes via H3R2me2s. PRMT5 is responsible for activating DNA damage repair genes such as BRCA1/2, RAD51, and ATM [12]. Amplified PRMT5 activity drives the expression and activity of key oncogenes (c-MYC, Cyclin D1, Notch1) while silencing expression and activity of tumor suppressors (ST7, RBL2, and p53). PRMT5 is involved in the regulation of cellular growth and differentiation, cell signaling cascades, and chromatin [13,14,15]. It also plays a significant role in the cell cycle and cell stemness [16,17]. PRMT5 suppression downregulated various genes involved in stem cell maintenance (PAX3, CDH4, KIF1A, and UCN2) as well as oncogenesis (CDH4, MMP14, and ARPC1B) [18].

### 2.2. Expression of PRMT5 in Diverse Cancers

Higher level of PRMT5 is associated with diverse cancers. The role of PRMT5 in tumorigenesis and its potential as a therapeutic target in cancer have been extensively reviewed [19]. In most tumor types, higher PRMT5 expression is directly linked to poor prognosis and survival outcomes [20]. Increased PRMT5 expression has been correlated with higher cancer growth in hepatocellular carcinoma, glioblastoma, lung, ovary and breast cancers [21,22,23,24,25,26,27,28]. Higher PRMT5 expression and symmetric methylation mark H4R3 were observed in lung cancer cell lines [23]. Cytoplasmic and nuclear PRMT5 expression levels were higher in non-small cell lung cancer (NSCLC) [24]. STAT3 was identified as a PRMT5 substrate in NSCLC cells. Arginine methylation at 609 increases STAT3 expression, leading to maintenance of cancer stem cells and cancer cell growth [25]. PRMT5 induces angiogenesis and EMT by regulating HIF-1α/VEGFR/Akt/eNOS signaling axis [26]. In both colon cancer and hepatocellular carcinoma, PRMT5 is highly expressed, and its downregulation reduces cell migration by decreasing MMP-2 expression [27]. Increased expression level of PRMT5 in colorectal cancer patients promoted cancer growth and migration. PRMT5 physically interacted with MCM7, and its level was also higher in colorectal cancer [28]. Various studies have reported that PRMT5 was highly expressed in glioblastoma (GBM), and its expression reduces the overall survival of the patients [29,30]. PRMT5 plays a crucial role in maintaining the self-renewal of mature and undifferentiated GBM tumor cells [31]. A specific PRMT5 inhibitor CMP5, was identified for GBM and found to induce apoptosis of differentiated cells and promote self-renewal of patient-derived GBM neurospheres [32].

## 3. PRMT5 Expression in Breast Cancer

Various reports suggest that PRMT5 regulates signaling pathways that affect breast tumor potential [33]. PRMT5 protein expression levels were higher in various breast cancer cell lines, including estrogen receptor-positive (ER^+^) and triple-negative breast cancer (TNBC) cell lines, compared to normal human mammary epithelial cells [34,35,36]. PRMT5 expression levels were higher in breast cancer samples than in normal tissues [34,35,36]. Breast cancer patients with higher PRMT5 expression exhibited poor prognosis, larger tumor size, and higher metastasis potential [35]. Further, higher PRMT5 levels were associated with increased Ki-67 expression in breast tumors [37]. Elevated Ki-67 expression is associated with more aggressive breast cancer [38]. PRMT5 regulates different breast cancer signaling pathways. The influence of PRMT5 on cell cycle regulation and cancer stem cell maintenance has been reported [34,35,36]. PRMT5 inhibition decreased cell growth by reducing the G1-to-S cell cycle transition in ER^+^/RBKO breast cancer and in both p53 mutant (T47D) and p53 wild-type (MCF-7) CDK4/6-resistant cells lacking RB [35]. PRMT5 enhanced the WNT/β-CATENIN pathway by reducing WNT antagonists DKK1 and DKK3 [36]. PRMT5-induced WNT/β-CATENIN signaling promotes proliferation in breast cancer. A higher level of PRMT5 increased breast cancer cell stemness, associated with cell markers CD24^−^/CD44^+^, and higher expression levels of c-MYC, OCT4/A, and Krüppel-like factor 4 (KLF4). Doxorubicin resistance was also associated with higher PRMT5 expression [34]. Glycolysis serves as an energy source for breast cancer growth [39]. Increased expression of PRMT5 is linked to KLF4 accumulation in all types of breast cancer [40]. KLF4 is a transcription factor identified as a tumor suppressor in gastrointestinal, esophageal, lung, and pancreatic cancer, whereas it acts as a tumor inducer in breast cancer and squamous cell carcinoma [41]. Studies showed that KLF4 methylation by PRMT5 reduces KLF4 ubiquitination and stabilizes its expression level by blocking its degradation. Further, mutation of PRMT5 methylation sites in KLF4 resulted in reduced breast cancer progression [40].

Downregulation of PRMT5 by si-PRMT5 treatment in ER^+^ and HER2^+^ breast cancer cell lines resulted in decreased glycolysis, reduced expression of Glut1, HK2, LDH-A, and NF-κBp65, and upregulation of LXRα expression [35]. Furthermore, studies suggested that PRMT5-induced tumor formation and metastasis is mediated by LXRα/NF-κBp65 [35]. PRMT5 regulation of epithelial–mesenchymal transition (EMT) was mediated by lysine-specific demethylase 1 (LSD1), which upregulates Slug expression and promotes a more advanced EMT and breast cancer metastasis [42]. Another study demonstrated that either shRNA-mediated depletion of PRMT5 or treatment with the selective inhibitor pemrametostat inhibited the growth of ER^+^/RB-deficient breast cancer cells [43].

### 3.1. PRMT5 Involvement in Breast Cancer Chemoresistance

Multiple studies have reported that elevated PRMT5 expression is associated with enhanced breast tumor growth, decreased survival, and sustained self-renewal of breast cancer stem cells [44,45]. PRMT5-mediated efficient DNA damage repair in these stem cells contributes to the development of resistance to chemotherapy and radiation therapy in breast cancer [46]. Hence, a combination of DNA-damaging chemotherapies with PRMT5 inhibitors may abolish breast cancer stem cells and reduce the drug resistance [46]. Another study also identified that a higher level of PRMT5 promotes breast cancer stemness and doxorubicin resistance via the regulation of OCT4/A, c-MYC, and KLF4 [34]. Although CDK4/6 inhibitors reduce tumor growth and prolong survival in ER^+^ breast cancer, patients ultimately develop resistance to these agents. A recent study reported that combining pemrametostat, a PRMT5 inhibitor, with fulvestrant, an ER antagonist, synergistically blocks the G1-to-S transition and effectively suppresses the growth of ER^+^/RB-deficient tumors [43].

### 3.2. PRMT5 and Triple-Negative Breast Cancer (TNBC)

TNBC is one of the most aggressive forms of breast cancer, characterized by a high metastatic potential, limited treatment options, and lower survival rates compared to other breast cancer subtypes. Compared to other breast cancer subtypes, TNBC develops chemotherapy resistance very quickly. When comparing TNBC with other breast cancer subtypes, PRMT5 mRNA expression levels are comparable to those observed in luminal and HER2^+^ breast cancers. However, differences in the subcellular localization of PRMT5 have been observed in TNBC relative to other breast cancer subtypes, with TNBC samples showing lower nuclear levels expression [47]. Another study using tissue microarray analysis demonstrated that PRMT5 is moderately expressed across various breast cancer tissues, with significantly higher expression observed in TNBC [48]. Similarly, studies using breast cancer cell lines correlated these findings, showing that the normal epithelial cell line MCF10A expresses low levels of PRMT5, whereas SKBR3 and BT474 exhibit moderate expression, and MDA-MB-231 shows high PRMT5 expression [48]. In the case of chemotherapy resistance, specifically in nanoparticle albumin-bound paclitaxel (Nab-PTX)-resistant cells, total PRMT5 levels remained unchanged compared to wild-type cells. However, PRMT5 protein expression was higher cytoplasm, but reduced in the nuclear fraction [49]. Further studies showed that there is an association exist between higher PRMT5 expression with lower survival of TNBC patients [47,48].

Long-term steroid treatment has been used to mitigate side effects associated with chemo- and immunotherapy; however, studies have been limited in demonstrating the impact of steroids on cancer growth and metastasis. Available reports indicate that PRMT5 methylates the glucocorticoid receptor (GR) and that this modification regulates its activity in breast cancer cells [50]. A recent study using a zebrafish model demonstrated that, in addition to its methyltransferase activity, PRMT5 regulates the GR action by recruiting phospho-HP1γ and RNA polymerase II. The GR/PRMT5/HP1γ complex modulates the transcription of glucocorticoid target genes, promoting increased tumor metastasis. Notably, TNBC tumors exposed to chemotherapy exhibited enhanced GR–PRMT5 interaction, potentially increasing the risk of metastatic progression [51]. This study provides another mechanism behind the action of PRMT5 in TNBC metastasis.

### 3.3. PRMT5 Enhances Stemness and Tumor Growth in TNBC

Role of PRMT5 on cancer stem cell maintenance has been reported among different breast cancers including TNBC. EPZ015666, a specific inhibitor of PRMT5, reduced the PRMT5-specific methylation, such as H3R8me2 and H4R3me2s. EPZ015666 inhibited the growth of different TNBC cell lines, reduced cancer stemness, and inhibited tumor growth in the TNBC patient-derived xenograft model [47]. The IC_50_ values of EPZ015666 were 1 µmol/L for the MDA-MB-453 cell line and 2.2 µmol/L for the MDA-MB-468 cell line. Additionally, four weeks of EPZ015666 treatment reduced TNBC tumor growth by 39% without causing toxicity [47]. TNBC is associated with methylthioadenosine phosphorylase (MTAP) deficiency. Oxime ether derivatives have been identified as second-generation PRMT5 inhibitors. The second-generation PRMT5 inhibitors showed most effectiveness towards MTAP deficient cells. These inhibitors exhibited cytotoxicity against TNBC in nanomolar levels (IC_50_-4.4 nM) and exhibited anti-tumor efficacy in an MTAP-null MDA-MB-231 xenograft model [52]. PRMT5 inhibitor, compound 5 (CMP5), reduces PRMT5 methylation of H3R8 and H4R3 histones in the DKK1 and DKK3 promoter region and decreases cyclin D1 and survivin expression in TNBC cell lines [36].

**Figure 1:**
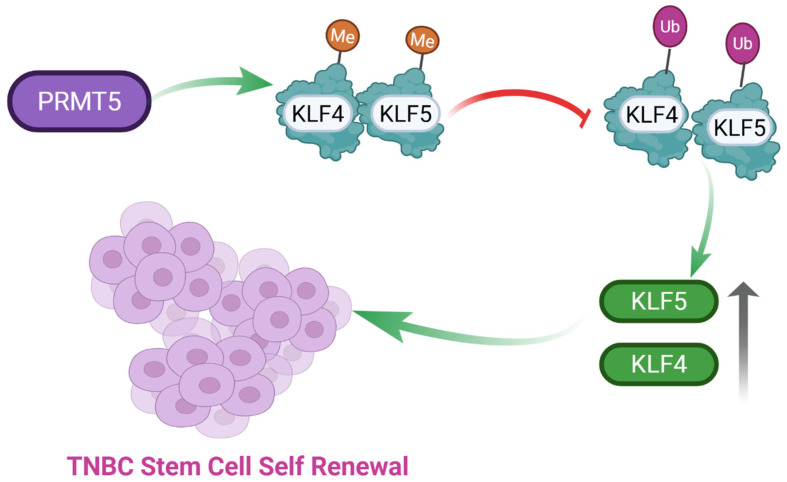
PRMT5-dependent methylation of KLF4 and KLF5 increases the stability of their protein expression levels.

Various downstream signaling mechanisms have been identified that correlate with PRMT5’s higher expression level and the aggressive nature of TNBC. PRMT5 plays a critical role in regulating the expression of the transcription factor KLF4. PRMT5 residues E483 and E489 interact with KLF4 at arginine positions 374, 376, and 377, which are located near its carboxy-terminal region [40]. Specifically, PRMT5-mediated methylation of KLF4 enhances the stability of the protein, thereby maintaining its expression levels [40]. KFL4 and PRMT5 interaction induces cancer cell stem cell self-renewal, epithelial–mesenchymal transition (EMT), and metastasis [40]. A strong association exists between PRMT5 and KLF5 in basal-like breast cancer, a TNBC subtype. KLF4/KLF5 methylation by PRMT5 reduces protein ubiquitination and degradation. Higher KLF4/KLF5 expression plays a significant role in breast cancer stemness [Figure 1]. Hence, PRMT5 increases TNBC breast cancer stem cell maintenance and cancer growth by stabilizing KLF4/KLF5 [40,53].

**Figure 2:**
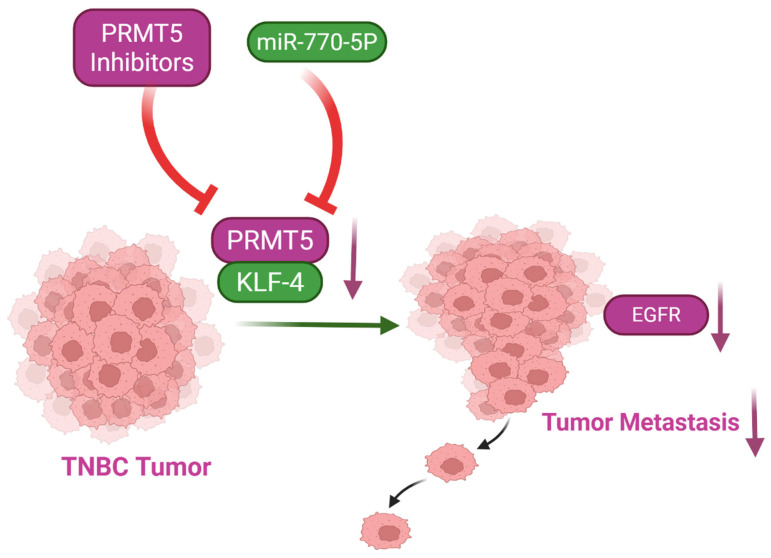
The oncogenic PRMT5–KLF4 signaling axis promotes tumor metastasis and is negatively regulated by PRMT5 inhibitors and miR-770-5P.

Noyan et al. studied the regulation of the PRMT5/KLF4 axis in TNBC and demonstrated that miR-770-5p inhibits PRMT5 expression and reduces KLF4 stability. The blockage of the PRMT5/KLF4 axis reduces EGFR signaling in TNBC. KLF4 is a major regulator of EGFR expressions in TNBC [54]. KLF4 inhibits EGFR gene expression, leading to reduced growth of aggressive tumors [55]. miR-770-5p is considered as tumor suppressor and its expression is significantly lower in breast cancer cell lines and patient tumor samples [54]. Methylation of KLF4 by PRMT5 is associated with higher EGFR expression and promotes tumor progression [Figure 2]. Moreover, it has been established that KLF4 plays a critical role in TNBC stem cell maintenance [53] [Figure 1]. KLF4/PRMT5 axis action varies based on breast cancer cell types; significant expression of KLF4 is detected in TNBC cell lines, a moderate level in HER2 breast cancer (SKBR3 and BT474), and the least in normal mammary epithelial cell lines MCF10A [48,55]. Similar patterns of KLF4 and PRMT5 expressions were observed in breast cancer patients, the highest in TNBC patients compared to other breast cancer patients [48]. Zhou et al. developed WX2–43, a drug that enhances the degradation of PRMT5/KLF4 axis in TNBC [48]. Inhibition of PRMT5-KLF4 oncogenic axis by WX2–43 may inhibit TNBC stem cell proliferation and can be considered a novel strategy for TNBC treatment. Treatment with 40 mg/kg or 80 mg/kg WX2-43 reduced TNBC tumor burden by approximately 60% and 40%, respectively [48]. Wang et al. identified another inhibitor PJ-68, which blocks the PRMT5-KLF4 axis and has shown significant tumor growth inhibition in the TNBC model [53].

### 3.4. PRMT5 Inhibitors Induces Apoptosis in TNBC

Various *in vitro* and *in vivo* studies have reported that PRMT5 plays a key role in inducing resistance to chemo/immunotherapy in TNBC. Several epigenetic targets, including bromodomain family proteins and histone methylating enzymes EZH2, G9a, and PRMT5, were identified in a paclitaxel-resistant TNBC cell line (MDA-MB-436) [56]. Of these, PRMT inhibitors showed the greatest anti-cancer activity in paclitaxel-resistant TNBC cells. Inhibition of PRMT1 or PRMT5 alone or in combination was effective in inhibiting the growth of paclitaxel-resistant cells. Further, this study showed that the PRMT5 inhibitors (GSK595/LLY283) induced apoptosis in paclitaxel-resistant cells by reducing AURKB expression and associated chromosomal instability [Table 1] [56]. Inhibition of PRMT5 causes premature termination of AURKB translation by promoting retention of the fifth intron in paclitaxel-resistant cells, but not in wild-type cells [56]. Nab-PTX-resistant TNBC cell lines showed higher cytoplasmic PRMT5 expression, increased autophagic vacuoles. and exhibited enhanced dimethylation of UNC-51-like kinase 1 (ULK1). ULK1 is a substrate of PRMT5 and an initiator of autophagic flux. PRMT5-mediated dimethylation of ULK1 enhances its activity. Further, inhibition of PRMT5 by shRNA in Nab-PTX-resistant cell lines reduced autophagic vacuoles, dimethylation of ULK1, increased sensitivity to Nab-PTX, and increased apoptosis. This study suggested that cytosolic PRMT5 levels play a significant role in the development of chemoresistance in TNBC [49].

**Figure 3:**
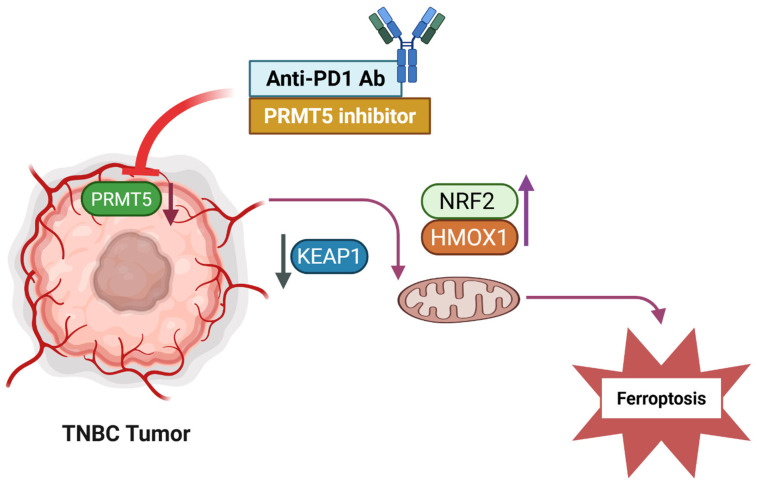
PRMT5 regulates ferroptosis via NRF2/HMOX1 pathway.

Ferroptosis is a regulated cell death process, and recent studies have reported that TNBC cells undergo ferroptosis, which can serve as an indicator of therapeutic response. Studies using MDA-MB-231 and HCC1937 cell lines showed that ectopic expression of PRMT5 reduced ferroptosis, significantly reduced NRF2 expression, and decreased HMOX1 expression [57]. The NRF2/HMOX1 pathway plays a significant role in regulating ferroptosis [Figure 3]. Furthermore, this study demonstrated that the combined treatment with a checkpoint inhibitor anti-PD-1 antibody and a PRMT5 inhibitor in a TNBC mouse model significantly inhibited tumor growth, decreased KEAP1 expression, and increased NRF2 and HMOX1 compared to immunotherapy alone. This study indicated that PRMT5 confers resistance to immunotherapy in TNBC and suggested that PRMT5 inhibitors potentiate the therapeutic efficacy of immunotherapy against TNBC [57] [Figure 3]. Guo et al. used PROTAC (Proteolysis Targeting Chimeras) technology to develop a PRMT5 inhibitor against TNBC. PROTAC uses the ubiquitin-proteasome system (UPS) to degrade PRMT5 [58]. This study identified YZ-836P, which decreases the expression of PRMT5 and KLF5.

YZ-836P inhibited the growth of TNBC cell lines HCC1806 and HCC1937, with IC_50_ values of 2.1 µM and 1.0 µM, respectively. Additionally, YZ-836P reduced patient-derived organoid formation and suppressed tumor growth in TNBC-bearing animals [Table 1] [58].

## 4. Combination of PRMT5 Inhibitors and Anti-Cancer Agents Against TNBC

Various biological processes, such as stem cell self-renewal, cell proliferation, and apoptosis, can be regulated by KLF4. An association between KLF4 and PRMT5 has been reported in TNBC [40,53]. A breast cancer tissue array analysis revealed increased protein expression of PRMT5 and KLF4 in TNBC tumors compared with other breast cancer subtypes, which may contribute to the initiation and progression of mammary tumors. Studies suggest that downregulation of PRMT5 reduces KLF4 expression and sensitizes MDA-MB-231 cells to chemotherapeutic drugs doxorubicin and cisplatin. KLF4 methylation by PRMT5 regulates TNBC cell survival and cancer stem cell maintenance. Further, WX2-43 was identified as an inhibitor that ablates the PRMT5-KLF4 interaction and inhibits TNBC stemness and progression [48].

Poly (ADP-ribose) polymerase (PARP) inhibitors are used specifically to treat TNBC patients with BRCA1 or BRCA2 mutations. A study was carried out to examine the combined effects of PARP inhibitors and PRMT5 inhibitors (GSK3326595 and TNG908) in a TNBC cell line with various BRCA1/2 statuses. The highest synergistic effect was observed in BRCA1/2 wild-type breast cancer cell lines (MDA-MB-468 and MDA-MB-231), whereas the synergistic effect was lower in the BRCA1 mutant breast cancer cell lines (SUM149PT and MDA-MB-436). This study showed that the synergistic effect of PARP inhibitors and PRMT5 inhibitors in TNBC cell lines is independent of the BRCA1/2 status and/or the MTAP status [59]. Another study used the same approach, combining the PARP inhibitor, olaparib, with the PRMT5 inhibitor EPZ015666. A combination of these inhibitors increases the survival of a TNBC BRCA-mutant patient-derived xenograft (PDX) model BCM-7482 [60]. Forty-two percent of the experimental mice treated with olaparib and EPZ015666 survived for seven months [60]. A recent study using patient-derived organoid (PDO) and xenograft (PDX) models suggested that combining olaparib with the PRMT5 inhibitor LLY-283 synergistically inhibited TNBC tumor formation. The study also demonstrated that immunotherapy with an anti-PD-1 antibody, when combined with olaparib and the PRMT5 inhibitor, showed greater efficacy than either dual-agent therapy or monotherapy [61]. Further, this study identified that interferon signaling can serve as a biomarker for PRMT5 inhibitor effectiveness [61]. The study by Zhang et al. showed that simultaneous blockade of PRMT1 (via GSK3368715) and PRMT5 (via GSK3235025) decreases ERCC1 expression and synergistically boosts PARP inhibitor activity in ovarian and TNBC models [21].

The PRMT5 inhibitor EPZ015938 showed varying effects in TNBC cell lines, with the maximum antiproliferative action observed in the HCC38 cell line (IC_50_ = 21.9 nM), followed by MDA-MB-453 (IC_50_ = 109.4 nM) and MDA-MB-468 (IC_50_ = 319.3 nM). Further, it was observed that PRMT5 inhibition synergized with various chemo drugs; a greater effect was noted with cisplatin, followed by doxorubicin or camptothecin, to impair TNBC cell proliferation [62]. More than half of TNBC patients exhibit higher EGFR expression [63]. Hence, studies were carried out to assess the combination of EPZ015938 and erlotinib. Erlotinib synergized with EPZ015938 to inhibit proliferation in BT20 and MDA-MB-468 cells, both of which display elevated EGFR expression [62].

Epithelial–mesenchymal transition plays a significant role in TNBC metastasis and chemotherapy resistance. Zhang et al. identified that PRMT5 and LSD1 epigenetically induce Slug expression, which leads to higher EMT process. Hence, pre-clinical studies were carried out to assess the anticancer activity of both PRMT5 and LSD1 Inhibitors. The tumor volume and lung metastasis were significantly decreased by the combined treatment with the PRMT5 inhibitor EPZ015666 and the LSD1 inhibitor SP2509 in the MDA-MB-231 xenograft model compared to the individual treatment with either EPZ015666 or SP2509 [42]. Tadalafil, an FDA-approved PDE5 inhibitor used for erectile dysfunction and pulmonary arterial hypertension, has also been identified as a potent PRMT5 inhibitor [64]. Tadalafil enhanced the effect of doxorubicin in MDA-MB-231 cells, which harbor wild-type BRCA1, but not in BRCA1-mutant HCC1937 cells. It additionally reduced doxorubicin resistance in MDA-MB-231 cells. A synergistic antitumor effect of tadalafil combined with doxorubicin was further demonstrated in MDA-MB-231 xenografts and in wild-type BRCA1 PDX mouse models. This synergy is attributed to tadalafil’s ability to inhibit RNA m6A methylation of BRCA1 by promoting ALKBH5 nuclear localization [65].

Activation of AKT induces oncogenic signaling in TNBC. Monotherapy with an AKT inhibitor did not show significant anti-tumor action in aggressive breast cancer patients. Yin et al. reported that PRMT5 dimethylates R391 of AKT and enhances its activity. Combined treatment of a PRMT5 inhibitor, GSK3326595, and an AKT inhibitor, MK2206, in three TNBC cell lines, MDA-MB-231, MDA-MB-468, and BT-549, showed a synergistic effect in inducing apoptosis. GSK3326595 also increased the action of etoposide and cisplatin in TNBC cells [66]. Another cell culture study demonstrated that combining the GSK3326595 with either doxorubicin or carboplatin markedly reduced proliferation and spheroid formation in MDA-MB-231 cells [67]. In a TNBC-patient-derived xenograft model, a triple combination therapy consisting of the PRMT5 inhibitor GSK3326595, the PRMT1 inhibitor GSK3368715, and carboplatin resulted in significant inhibition of tumor growth [67]. Table 2 summarizes studies evaluating PRMT5 inhibitors in combination with other therapeutic agents.

### 4.1. Effect of PRMT5 Inhibitors for Diverse Cancers

Various PRMT5 inhibitors showed efficacy against various cancers. The PRMT5 inhibitor PRT382 demonstrated anticancer activity both as a monotherapy and in combination with the BCL-2 inhibitor venetoclax in mantle cell lymphoma [68,69]. Additionally, another PRMT5 inhibitor, LLY-283, exhibited antitumor efficacy in melanoma xenograft models [70], as well as in glioblastoma [71]. GSK3326595 was effective for both p53 wild-type B-cell acute lymphoblastic leukemia [72], and for lung cancer xenograft model [73]. EPZ015666 showed anti-cancer effect against mantle cell lymphoma (MCL) cell lines [74] and retinoblastoma tumor moue model [75]. A study using glioblastoma cells found that PRMT5 expression was increased with rapamycin, an mTOR inhibitor. Blockade of PRMT5 expression in GBM cells (LN229, LN18, GBM6, and GBM39) and in patient-derived cell lines using the siRNA approach increased sensitivity to mTOR inhibitors (rapamycin and PP242). Both cyclin D1 and c-MYC IRES activities were decreased in PRMT5 downregulation using PRMT5 siRNA and following mTOR inhibitor (PP242) exposure [76]. Another study using patient-derived glioblastoma cancer stem cells showed that PRMT5 inhibition is associated with significant changes in the expression levels of several genes involved in cell cycle progression, leading to reduced disease progression and resistance to radiotherapy and chemotherapy [71]. Neuroendocrine differentiation (NED) is considered a phenotype change associated with chemotherapy resistance in NSCLC. A study by Shen et al. found higher levels of PRMT5 mRNA expression in etoposide-treated A549 cells. Downregulation of PRMT5 reduced NED and significantly enhanced the efficacy of etoposide in the A549 cell line [77]. There have also been reports on the role of PRMT5 in immunotherapy resistance. PRMT5 suppression increased the anti-cancer action of immunotherapy in cervical cancer and melanoma [78,79]. Targeting PRMT5 and PD-L1 has been shown to enhance anti-tumor activity in lung cancer. Loss of PRMT5 further increases the therapeutic efficacy of PD-L1 antibody treatment [80].

### 4.2. PRMT5 Inhibitors in Clinical Trial

PRMT5 inhibitors have not yet received FDA approval for any cancer types. Several clinical trial details on PRMT5 inhibitors across a range of cancers are available [Table 3] [81]. However, very few clinical trials were conducted for breast cancer. A Clinical trial was carried out using GSK3326595, using early-stage breast Cancer (OTT-19-06) (NCT04676516) patients. This study included 60 hormone-receptor-positive breast cancer patients who received GSK3326595 before breast surgery. No reports are publicly available from this trial yet. However, the same drug was used for two trials for other cancer types. A phase I/II clinical trial (NCT03614728) with GSK3326595 (400 or 300 mg) monotherapy was carried out for adults with relapsed/refractory myeloid neoplasms. This study demonstrated limited anti-cancer activity of GSK3326595 in heavily pretreated myeloid cancer patients, with a clinical benefit rate observed in 17% of participants. Common adverse effects associated with GSK3326595 treatment include nausea, fatigue, and changes in taste perception [82]. A multicenter phase I trial (NCT02783300) evaluated GSK3326595 as both monotherapy and in combination with pembrolizumab across several solid cancer types and non-Hodgkin lymphoma. As a single agent, GSK3326595 demonstrated modest anti-tumor activity in multiple malignancies including non-Hodgkin lymphoma and HR^+^ breast cancer. In contrast, the combination of GSK3326595 with pembrolizumab did not produce observable anti-cancer responses. Additionally, the study identified symmetrical dimethylarginine (SDMA) as a pharmacodynamic marker of PRMT5 inhibition in patients with diverse cancers. No serious adverse effects were reported with GSK3326595; the drug was well tolerated. The observed adverse effects, such as nausea, fatigue, and anemia, were reversible [83]. Another PRMT5 inhibitor, PF-06939999, was evaluated for its clinical response, safety, and tolerability in 28 patients with solid tumors (NCT03854227). The dosage used in the study was 0.5, 4, 6, or 8 mg once daily, or 0.5, 1, 2, 4, or 6 mg twice daily. The study used SDMA as the marker for PRMT5 inhibition. The study outcomes demonstrated that a daily dose of 6 mg PF-06939999 was optimal for achieving a 78% reduction in plasma SDMA, reflecting maximal inhibition of PRMT5. Previous studies suggested that complete tumor inhibition was associated with a 78% decrease in SDMA levels [84]. Moreover, the platelet reduction observed at 6 mg daily dose was in an acceptable range [84]. Another report from the same phase I clinical study (NCT03854227) reported that after 15 days of treatment, plasma SDMA levels showed a sustained reduction of 58–88%. Adverse events such as anemia, thrombocytopenia (22%), and fatigue were reported [85]. Another PRMT5 inhibitor, JNJ-64619178, was evaluated in patients with advanced malignant solid tumors. The tumor types were adenoid cystic carcinoma, prostate, pancreatic, and uveal melanoma. Deceased levels of SDMA were observed in all patients, confirming PRMT5 inhibition. Anti-tumor activity was most observed in adenoid cystic carcinoma patients, with a median progression-free survival of 19.1 months and an overall response rate (ORR) of 11.5% (3 of 26). Anemia, thrombocytopenia, fatigue, and nausea were the adverse effects associated with a higher dose of JNJ-64619178 [86]. A Phase 1 multicenter study evaluated JNJ-64619178 in 24 patients with lower-risk, transfusion-dependent MDS harboring SF3B1 mutations. The trial identified a tolerable daily dose of 0.5 mg and documented a 70–80% reduction in plasma SDMA levels. However, no clinical benefit was achieved, neither hematologic improvement nor transfusion independence. The patients experienced substantial hematologic toxicities, including neutropenia, thrombocytopenia, and anemia [87]. A phase I clinical study of PRT543 monotherapy was undertaken in patients with myelodysplastic neoplasms and secondary acute myeloid leukemia. The study results confirmed PRMT5 inhibition, with a 41.9% reduction in serum SDMA levels. PRT543 treatment demonstrated modest clinical efficacy in hematologic malignancies [88].

## 5. Future Perspectives

PRMT5 is highly expressed in most cancers, and it is associated with poor survival and patient outcome. The PRMT5 downstream signaling molecules may exhibit either oncogenic or tumor-suppressive effects. Further, the chemotherapy resistance related to higher PRMT5 expression induces cancer stemness and metastasis. Studies indicate that one mechanism underlying the association between higher PRMT5 expression and chemotherapy resistance is stem cell activation, mediated by OCT4/A, c-MYC, and KLF4 [34].

TNBC is one of the most aggressive breast cancers, and higher PRMT5 expression in TNBC reduces NRF2/HMOX1-mediated ferroptosis, a regulated cell death process, and thereby reduces patient survival. Higher PRMT5 expression is also associated with immunotherapy resistance in TNBC [57]. A TNBC animal model study showed that combined treatment with an anti-PD-1 antibody and a PRMT5 inhibitor increased NRF2 and HMOX1 expression and enhanced the anti-cancer activity of immunotherapy [57] [Figure 3]. Paclitaxel resistance can be overcome with a PRMT5 inhibitor, which reduces AURKB expression and chromosomal instability in TNBC [56]. KLF4 and KLF5 transcription factors are substrates of PRMT5; their methylation reduces protein ubiquitination, resulting in increased protein stability and expression [40,49] [Figure 1]. PRMT5 methylation also increases EGFR expression. These signaling changes lead to cancer stem cell self-renewal and aggressive TNBC [53,54]. miR-770-5p disrupts PRMT5/KLF4 association, thereby reducing EGFR expression levels [Figure 2]. Further, WX2-43, an inhibitor that disrupts PRMT5-KLF4 interaction, reduces TNBC stemness and progression [48]. Downregulation of PRMT5 and KLF4 showed an increased sensitivity of chemotherapy drugs in TNBC cells [48]. Elevated PRMT5 expression correlates with multiple signaling molecules that regulate TNBC stemness and metastatic progression [Figure 1 and Figure 2]. PRMT5 represents a promising therapeutic target for TNBC, as chemotherapy resistance is a key driver of metastasis in this disease. Recent studies indicate that targeting PRMT5 increases the chemosensitivity of TNBC cells by suppressing stem-like properties and reducing signaling pathways associated with metastasis.

There have been multiple reports on the combination of PRMT5 inhibitors and existing TNBC treatments. Treatment of PRMT5 inhibitor EPZ015938, together with chemotherapy drugs (cisplatin, doxorubicin, or camptothecin) and EGFR inhibitor (erlotinib), exhibited synergistic anticancer action against TNBC cell lines [62]. A synergistic anticancer effect was observed with PARP inhibitors and PRMT5 inhibitors (GSK3326595 and TNG908) in wild-type BRCA1/2 status. However, the combination of a PARP inhibitor and EPZ015666 (a PRMT5 inhibitor) increases survival in a TNBC BRCA-mutant xenograft model [60]. Another study showed that the combination of EPZ015666 and the LSD1 inhibitor SP2509 reduced TNBC tumor volume and metastasis [42]. Tadalafil (PRMT5 inhibitor) enhanced the effect of doxorubicin in wild-type BRCA1 cells [65]. Combined treatment with GSK3326595 showed synergistic anticancer activity against TNBC cells when combined with the AKT inhibitor MK2206 or the chemotherapy drug etoposide [66]. Tadalafil, an FDA-approved PDE5 inhibitor, is considered a PRMT5 inhibitor and enhances the doxorubicin anticancer action in the wild-type BRCA1 TNBC xenograft mouse model [65]. Collectively, these findings suggest that PRMT5 inhibitors, particularly when combined with other therapeutic agents, hold significant promise for the treatment of TNBC (Table 2). They highlight the potential of available PRMT5 inhibitors to modulate key signaling pathways involved in TNBC tumor growth and metastasis. Future clinical trials are needed to evaluate the efficacy of a novel PRMT5 inhibitors, alone or in combination with chemotherapy drugs/other oncogenic signaling pathway inhibitors in TNBC patients.

Several PRMT5 inhibitors are currently being evaluated in clinical trials across a range of cancers beyond breast cancer. One such agent, GSK3326595, has been tested in a clinical study involving 60 patients with hormone-receptor-positive breast cancer. This drug demonstrated efficacy in preclinical models of TNBC. At present, GSK3326595 is undergoing clinical evaluation both as monotherapy and in combination regimens for multiple malignancies, including relapsed or refractory myeloid neoplasms, non-Hodgkin lymphoma, and various solid tumors. However, the effectiveness of this drug remains limited or suboptimal across certain cancer types (Table 3). Other PRMT5 inhibitors, such as JNJ-64619178 and PF-06939999, are also being investigated in clinical trials for different types of cancer. These agents consistently show potent inhibition of PRMT5 activity, as indicated by decreased levels of SDMA. However, despite strong target engagement, their therapeutic efficacy varies across cancer types. This suggests that PRMT5 monotherapy may not be an effective option for cancer treatment. Therefore, combining PRMT5 inhibitors with other therapeutic agents may offer greater efficacy in treating cancer. Hematologic toxicity remains a commonly observed adverse effect among these PRMT5 inhibitors.

## 6. Conclusions

PRMT5 is overexpressed in multiple types of cancer, including breast cancer, and is considered a particularly relevant therapeutic target in TNBC compared to other breast cancer subtypes. Inhibition of PRMT5 using specific inhibitors has been shown to reduce cancer stemness and improve chemosensitivity. Moreover, PRMT5 inhibitors demonstrate synergistic effects when combined with other therapeutic agents, leading to enhanced suppression of tumor growth and metastasis. Clinical evaluation of the therapeutic potential of PRMT5 inhibitors in combination with chemotherapy or immunotherapy for TNBC may offer a promising targeted strategy for treating this aggressive breast cancer.

## Figures and Tables

**Table 1:** Effect of PRMT5 inhibition in TNBC cells and xenograft models.

PRMT5 Inhibition	TNBC Model	Signaling Molecule	Effect	Reference
Inhibitors-GSK595/LLY283	Paclitaxel-resistant MDA-MB-436/MDA-MB-231	↓ AURKB	↑ Apoptosis	[56]
PRMT5 shRNA	Nanoparticle albumin-bound paclitaxel resistant MDA-MB-231/MDA-MB-468	↓ ULK-1	↑ Apoptosis	[49]
GSK3326595 & Anti-PD-1 antibody	TNBC mouse model	↓ KEAP1 ↑ NRF2, HMOX1	↓ Tumor growth	[57]
YZ-836P	TNBC patient-derived organoids and HCC1806 Xenografts mice model	↓ KLF5	↓ Organoid formation & Tumor growth	[58]

Arrows are needed, it indicates decrease or increase the expression levels.

**Table 2:** Synergistic anti-cancer effects of PRMT5 inhibitors combined with other therapeutic agents in TNBC.

PRMT5 Inhibitors	Anti-Cancer Agents	TNBC Model	TNBC Signaling Molecule	Reference
WX2-43	Doxorubicin & Cisplatin	MDA-MB-231 xenograft model	↓ KLF4	[48]
GSK3326595	Talazoparib (PARP inhibitor)	MDA-MB-468 & MDA-MB-231	↑ BRCA1, BRCA2 RAD50	[59]
TNG908	Talazoparib	MDA-MB-468 (MTAP WT) & MDA-MB-231 (MTAPnull)		[59]
EPZ015666	Olaparib (PARP inhibitor)	BT549 & BRCA-mutant PDX model	↓ ATR & ATR pS428	[60]
LLY-283	Olaparib	Patient-derived organoid and PDX models	↑ Interferon	[61]
GSK3368715, GSK3235025	Olaparib	MDA-MB-231 & MDA-MB-468	↓ ERCC1	[21]
EPZ015938	Cisplatin, Doxorubicin Camptothecin	BT20, MDA-MB-453 and MDA-MB-468		[62]
EPZ015938	Erlotinib, Neratinib	BT20 and MDA-MB-468		[62]
EPZ015666	SP2509 (LSD1 inhibitor)	MDA-MB-231 Xenograft model	↑ E-cadherin ↓ Vimentin	[42]
Tadalafil	Doxorubicin	MDA-MB-231 Xenografts & Wild-type BRCA1 PDX mouse models	ALKBH5 nuclear localization	[65]
GSK3326595	MK2206 (AKT inhibitor)	MDA-MB-231, MDA-MB-468, and BT-549,		[66]
GSK3326595	Doxorubicin, Carboplatin	MDA-MB-231		[67]

Arrows are needed, it indicates decrease or increase the expression levels.

**Table 3:** Clinical Trials Evaluating PRMT5 Inhibitors for Multiple Cancers. Abbreviations: AML, Acute Myeloid Leukemia; CMML, Chronic Myelomonocytic Leukemia; CRi, Complete Remission with Incomplete Blood Count Recovery; HR+, Hormone Receptor-Positive; MDS, Myelodysplastic Syndromes; MPN, Myeloproliferative Neoplasm; NHL, Non-Hodgkin Lymphoma; ORR, Overall Response Rate; PFS, Progression-Free Survival; PR, Partial Response; QD, once daily; R/R, relapsed/refractory; RP2D, Recommended Phase II Dose; SDMA, Symmetric Dimethylarginine.

PRMT5 Inhibitor	Clinical Trial/ Phase	Tumor Type/ Population	Treatment	Efficacy	Toxicities	Reference
GSK3326595	NCT04676516; phase II window-of-opportunity trial	Early-stage HR+ breast cancer; *n* = 60	Pre-surgical monotherapy	Results not publicly reported	Results not publicly reported	
GSK3326595	NCT03614728; phase I/II	R/R MDS, CMML, AML; *n* = 30	Oral monotherapy; 300–400 mg/daily	Limited activity; Clinical benefit rate (17% of patients had response)	Cytopenias, dysgeusia, fatigue, nausea	[82]
GSK3326595	NCT02783300; phase Ib/dose expansion	Solid tumors and Non-Hodgkin lymphoma NHL) *n* = 288	Monotherapy and combination with pembrolizumab also tested	Modest monotherapy activity; responses in NHL, and solid tumors. No responses with pembrolizumab	Fatigue, nausea, anemia, dysgeusia, thrombocytopenia	[83]
PF-06939999	NCT03854227 Phase I (first-in-patient study)	Solid tumor *n* = 28	Dose escalation (part 1) and dose expansion (part 2). recommended dose for expansion (RDE) 6 mg QD	78% reduction in plasma SDMA	Thrombocytopenia	[84]
PF-06939999	NCT03854227; phase I dose escalation/expansion	Advanced/metastatic solid tumors	Oral monotherapy; 6 mg QD selected as RP2D	SDMA reduced 58–88%; 3 confirmed PRs	Anemia28%, thrombocytopenia (22%), fatigue, neutropenia	[85]
JNJ-64619178	NCT03573310; phase I	Advanced solid tumors; *n* = 90	Recommended phase 2 doses—1.5 mg intermittently (2 weeks on/1 week off) and 1.0-mg once daily	ORR 5.6%; ACC ORR 11.5%; median PFS 19.1 months in ACC. Plasma SDMA reduced	Anemia, thrombocytopenia, fatigue, and nausea	[86]
JNJ-64619178		lower-risk, transfusion-dependent MDS harboring SF3B1 mutations	Dose identified- 0.5 mg QD	70–80% reduction in plasma SDMA- no clinical benefit	Neutropenia, thrombocytopenia, and anemia	[87]
PRT543	NCT03886831; phase Ib	MDS, AML, MDS/MPN overlap *n* = 40	Oral monotherapy; RP2D 35 mg daily, 5 days/week	Modest activity; HI/mCR in MDS/MDS-MPN and CRi in 1 AML patient; SDMA reduced 41.9%	Thrombocytopenia, nausea, fatigue, neutropenia	[88]

## Data Availability

No new data were created or analyzed in this study. Data sharing is not applicable to this article.
